# A Nasal Cavity Mucosal Melanoma Connected by Nasolacrimal Duct in a Patient with Multiple Co-morbidities: A Treatment Dilemma

**DOI:** 10.4021/wjon236e

**Published:** 2010-08-29

**Authors:** Ching-Feng Chou, Shy-Chyi Chin, Li-Yu Lee, I-How Chen, Chun-Ta Liao, Shiang-Fu Huang

**Affiliations:** a Department of Otolaryngology, Head and Neck Surgery, Chang Gung Memorial Hospital, Tao-Yuan, Taiwan; b Department of Diagnostic Radiology, Chang Gung Memorial Hospital, Chang Gung University, Tao-Yuan, Taiwan; c Department of Pathology, Chang Gung Memorial Hospital, Tao-Yuan, Taiwan; d Chang Gung University, Tao-Yuan, Taiwan

**Keywords:** Melanoma, Nasal cavity, Paranasal sinuses, Nasolacrimal duct

## Abstract

Nasal cavity is a rare site in melanoma and surgery plays important roles in its treatment. A mucosal melanoma penetrating through the nasolacriminal duct (NLD) into orbit in a patient with multiple co-morbidities poses some difficulties in its management. A 78 year-old man developed epiphora and ocular swelling in recent 1 week in his left eye. He had medical histories of hypertension, diabetes, chronic renal insufficiency and right middle cerebral artery infarction. Physical examination revealed left periorbital swelling, chemosis, lateral gaze impairment and a dark-colored mass in left nasal cavity. Mucosal melanoma was diagnosed by biopsy. Contrast enhanced T1-weighted MRI showed an enlarged hyperdense lesion in left nasal cavity which invades through the NLD and gets into left medial aspect of orbit with involvement of preseptal space and retrobulbar fat. The patient was treated with radiation therapy alone (450 cGY in 13 fractions) with partial response. Lung metastasis occurred 3 months later and the patient was alive with disease for 6 more months.Primary surgical resection has been the treatment of choice for mucosal melanoma. Radiotherapy in our patient was chosen for multiple co-morbidities. Neither local nor regional control was improved by this approach. Palliative chemotherapy in this situation could be the treatment of choice for patient’s better quality of life.

## Introduction

Over the past half century, the incidence of melanoma has increased more than 10-fold in America. Approximately 20% of all melanomas occur in the head and neck, with less than 10% from the mucous membranes of the aerodigestive tract. One-third to one-half of these mucosal melanomas arise in the nasal cavity or paranasal sinuses; the majority of the remainder are found in the oral cavity [1]. Overall, less than 1% of all melanomas arise in the nasal cavity or paranasal sinuses [2]. The anatomic constrains of nasal cavity pose some difficulties in the diagnosis and surgical treatments for melanomas in these locations. The survival for mucosal melanoma of the nasal cavity and paranasal sinuses is thus generally poor.

We here present a 78-year-old man with melanoma involving both nasal cavity and orbit. The tumor penetrates through the nasolacriminal duct (NLD) and poses some difficulties in its management.

## Case Report

**Figure 1:**
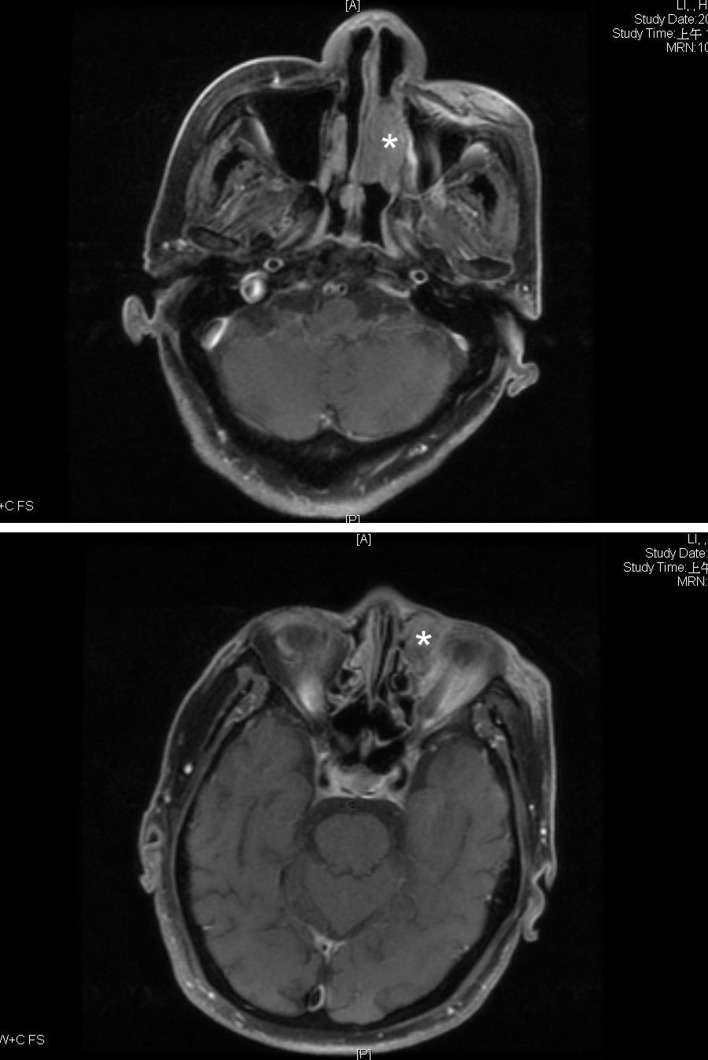
Contrast enhanced T1-weighted MRI showed (A) an enlarged hyperdense lesion in left nasal cavity (asterisk), (B) A hyperdense lesion in left medial aspect of orbit with involvement of preseptal space and retrobulbar fat were found on (asterisk).

**Figure 2:**
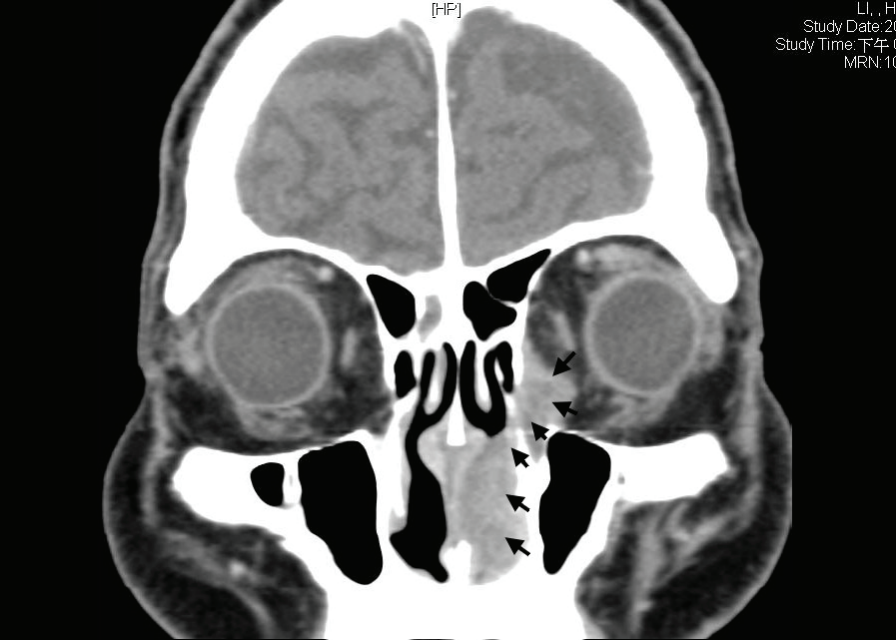
Contrast-enhanced CT demonstrated a dumbbell-shaped tumor in left medial canthal region and nasal cavity, connected by the nasolacrimal duct (arrowheads).

We here present a 78 year-old man with old stroke and under aspirin treatment presented with a 2-month history of nasal obstruction and epistaxis. He also developed epiphora and ocular swelling in recent 1 week in his left eye. Physical examination revealed left periorbital swelling, chemosis, lateral gaze impairment and a dark-colored mass in left nasal cavity. NLD obstruction was demonstrated by probing and irrigation. The nasal tumor was biopsied. Mucosal melanoma was diagnosed by identification of melanin in the tumor cells and positive staining with S-100. Contrast enhanced T1-weighted MRI demonstrated an enlarged hyperdense lesion in left nasal cavity (Fig. 1A, asterisk) and a hyperdense lesion in left medial aspect of orbit with involvement of preseptal space and retrobulbar fat (Fig. 1B, asterisk). Contrast-enhanced CT demonstrated a dumbbell-shaped tumor in left medial canthal region and nasal cavity, connected by the NLD (Fig. 2, arrowheads).

Maxillectomy with left orbit enucleation was not accepted by family due to high surgical and anesthetic risks. The patient had hypertension, diabetes and large right middle cerebral artery infarction and was treated with radiation therapy alone (450 cGY in 13 fractions). No chemotherapy was given due to chronic renal insufficiency. The tumor had partial response after radiation therapy. However, lung metastasis developed 3 months later. Chemotherapy and immunotherapy were given for the lung metastasis. But the tumor responded poorly to the therapy, patient deteriorated rapidly and expired 6 months later.

## Discussion

From the specimens of nasal tumor, we could clearly identify melanin. The diagnosis of this dumbbell-shaped tumor was a nasal cavity melanoma which invades into the orbit through the NLD. Mucosal melanoma in nasal cavity involves the orbit through the NLD was rare. The low incidence rate and the anatomic constrains of nasal cavity pose some difficulties in the diagnosis and surgical treatments for melanomas in these locations. The survival for mucosal melanoma of the nasal cavity and paranasal sinuses is thus generally poor.

Regarding the symptoms of nasal melanoma, unilateral obstruction and epistaxis accounted for 85-100% of symptoms [3]. Concomitant ocular symptoms including epiphora, ocular swelling occurred infrequently unless direct involvement of orbit by the nasal tumor. Two theories were proposed about the development of simultaneous orbit and nasal cavity melanoma. One is direct spread or spread via the blood vessels from the nasal cavity or the paranasal sinuses into the orbit. The other is the tumor invades directly into the orbit by destructing lamina papyracea or orbital floor. The spread of tumor through the NLDs instead of destructing bony structure was rarely reported [4], but it was apparent from both the CT can and MRI in our patient.

For mucosal melanomas in nasal cavity, primary surgical resection has been the treatment of choice. Adjuvant radiotherapy was reserved for unfavorable pathological features which included close or involved surgical margins, perineural invasion, large primary tumors, and nasal cavity or paranasal sinus location [5]. Recent radiobiologic studies and clinical studies have demonstrated initial complete and partial responses after high-dose per fraction radiotherapy [6]. In this patient, the tumor involves both the orbit and the nasal cavity. Surgical resection would include maxillectomy and orbital exenteration. Surgical risks in an old age and post-stroke patient were exceedingly high. Concerning the morbid sequelae, the patient received high-dose radiotherapy per fraction. However, the tumor persisted after radiation therapy (450 cGY in 13 fractions) therapy.

Three months later, lung metastasis developed 3 months in our patient after the treatments. Rapid clinical deterioration is typical in the setting of distant disease, a 5-months average survival following discovery of distant metastasis was reported [7]. In advanced stage patients, dacarbazine and/or cisplatin chemotherapy combined with IL-2 have been demonstrated a response rate of approximately 50%, with 10% of patients achieving a durable complete response [8]. However, the combination of chemotherapy and immunotherapy did not have durable effect in our patient.

In conclusion, a nasal cavity mucosal melanoma extends into orbit through NLD is rarely found clinically. The response of nasal cavity melanoma to radiotherapy alone was poor from our experience. Radical or maximal debulking surgery with post-operative radiotherapy or chemotherapy in this situation could be the treatment of choice for better tumor control.
